# Assessing uncertainty: A study of entropy measures for Burr XII distribution under progressive Type-II censoring

**DOI:** 10.1371/journal.pone.0329086

**Published:** 2025-08-08

**Authors:** Amal Helu, Hani Samawi

**Affiliations:** 1 Mathematics Department, The University of Jordan, Amman, Jordan; 2 Jiann-Ping Hsu College of Public Health, Georgia Southern University, Statesboro, Georgia, United States of America; The University of Warwick, UNITED KINGDOM OF GREAT BRITAIN AND NORTHERN IRELAND

## Abstract

This research study focuses on calculating five entropy measures (Shannon, Rényi, Havrda-Charvát, Arimoto, and Tsallis) for the Burr XII distribution, utilizing progressive Type-II censoring. The study derives maximum likelihood estimators for each entropy measure and constructs two-sided confidence intervals. A comprehensive simulation study evaluates the performance of these estimators across various sample sizes and parameter settings. The results demonstrate that the proposed methods achieve low bias and variance under different censoring schemes, with coverage probabilities consistently close to the nominal level. Additionally, an application to the Wisconsin Breast Cancer Database highlights the practical utility of the entropy estimators in distinguishing between benign and malignant cases. Among the measures evaluated, the Rényi, Havrda-Charvát entropy measures exhibited the most robust performance in both simulation and real life data analysis.

## 1 Introduction

Entropy, introduced by Shannon in 1948, is a fundamental concept for quantifying uncertainty in random variables. It measures the average information content of a variable, where higher entropy indicates greater uncertainty and a wider spread in the probability distribution. Conversely, lower entropy suggests a more concentrated distribution with reduced uncertainty. The concept of entropy has been widely applied across various scientific disciplines. For example, [1] explored its significance in the insurance industry, particularly in evaluating risk and the severity of extreme events, where greater entropy correlates with increased variability and potential losses. In reliability studies, [2–5] highlighted the relevance of entropy in assessing the uncertainty of failure distributions, noting that higher entropy is often associated with less reliable outcomes. Furthermore, entropy-based methodologies have been employed in fields such as neurobiology, statistics, cryptography, quantum computing, linguistics, and bioinformatics, as reported by [6–8]. These applications underscore the importance of entropy in both theoretical and applied research. Recent studies have also demonstrated the utility of entropy estimators in neuroscience and biomedical diagnosis, particularly in analyzing electrophysiological signals such as EEG data [9,10]. These applications highlight the versatility of entropy-based methods across diverse fields. However, the present study focuses on the methodological development and evaluation of entropy measures for lifetime data analysis under progressive Type-II censoring, particularly within the context of reliability modeling and failure time uncertainty.

### 1.1 The Burr XII distribution and its applications

The Burr XII distribution is an extremely useful model, especially when dealing with non-monotonic failure rates, such as unimodal or bathtub-shaped failure rates, which are widespread in reliability and biological research. Unlike the Weibull distribution, which may be the first choice for analysing monotonic failure rates due to its negatively and positively skewed density shape, the Burr XII distribution provides more flexibility in modeling non-monotonic failure rates.

The Burr XII model is crucial in reliability engineering as it predicts how long a system or component may last. In particular, this is true when only partial data is available as a result of early terminations of tests. Various medical outcomes can be modeled using this framework, commonly used in survival analysis. Finance uses the Burr XII distribution to analyze extreme events, such as financial crises, thereby making it an essential resource for managing risk. Likewise, environmental scientists use the Burr XII distribution to study complex patterns of nature, such as rainfall patterns, which are crucial for the effective management of natural resources. Moreover, the cumulative distribution function and the reliability function of the Burr XII distribution have closed forms, allowing for simplified percentile and likelihood calculations under censored data. For more on Burr XII and its applications, see [11–13].

Let X be a random variable from Burr XII(α,β) distribution. The probability density function (pdf) and cumulative distribution function (cdf) of *X* are given by:

$$f(x)=\alpha \beta x^{\beta -1}{\left(1+x^{\beta }\right)}^{-(\alpha +1)},\;x>0,\alpha ,\beta >0,$$ (1)

$$F(x)=1-{\left(1+x^{\beta }\right)}^{-\alpha },\;x>0,\alpha ,\beta >0,$$ (2)

where, α and β are the shape parameters. The corresponding survival function $\bar{F}(t)$ and hazard function *h*(*t*) are:

$$\bar{F}(t)={\left(1+t^{\beta }\right)}^{-\alpha },\;t>0,$$ (3)

$$h(t)=\alpha \beta t^{\beta -1}{\left(1+t^{\beta }\right)}^{-1},\;t>0.$$ (4)

**Fig 1:**
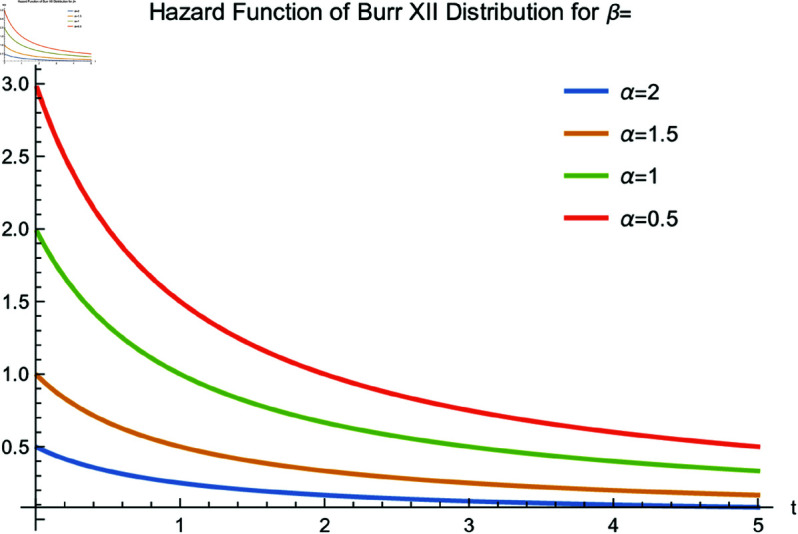
Hazard function of the Burr XII distribution for β=1 and various values of α.

**Fig 2:**
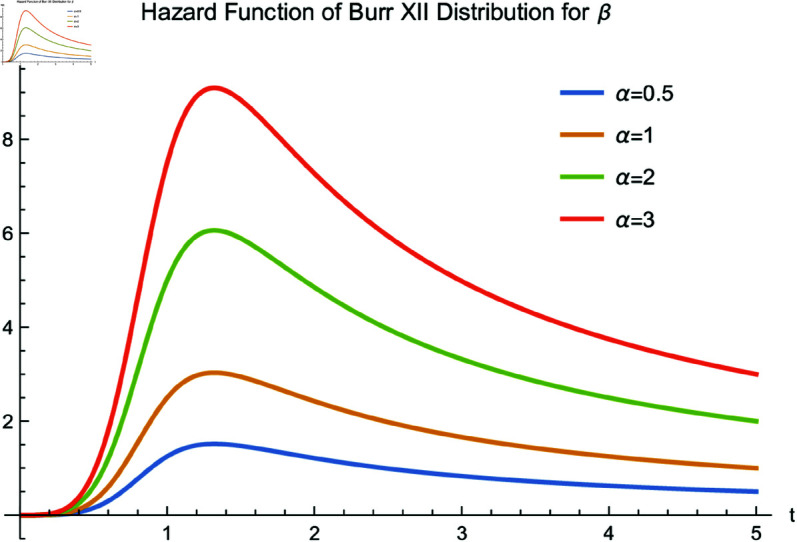
Hazard function of the Burr XII distribution for β=5 and various values of α.

Figs 1 and 2 illustrate the hazard function of the Burr XII distribution with different parameters. As shown, the hazard function is non-monotonic and can accommodate various shapes. According to [14,15], due to the shapes of *h*(*t*), the Burr XII distribution is widely used in quality control, reliability analysis, biological and life test studies, as well as in economics and industrial tests.

### 1.2 Entropy measures of the Burr XII distribution

**Shannon entropy:** Let *X* be a random variable with the pdf given in Eq (1). The Shannon entropy of the Burr XII distribution is defined as:


S=−E[logf(x)],

$$S=-\int_{0}^{\infty }f(x)\log f(x)\;dx,$$ (5)

$$S=-\left(\frac{1}{\beta }-1\right)\left[\psi (\alpha )+\gamma \right]+\frac{1}{\alpha }-\log(\alpha \beta )+1,$$ (6)

where, ψ(s) is the digamma function and γ is the Euler-Mascheroni constant γ≈0.5772.

Shannon entropy is one of the earliest and most commonly used entropy measures. This measure has proven effective in the study of communication systems. However, one significant disadvantage of the Shannon measure, particularly in the continuous case, is that it may be negative for certain probability distributions, complicating its interpretation as a measure of uncertainty. Various generalizations have been proposed to address the limitations of Shannon entropy.

**Rényi entropy:** [16] introduced a generalized entropy by extending the concepts of uncertainty and randomness. The Rényi entropy, which generalizes Shannon’s entropy, is parameterized by a single parameter, *p*. As *p* approaches unity, it converges to the familiar Shannon entropy. A notable property of Rényi entropy is that in algorithms requiring entropy maximization, Rényi’s entropy can be substituted directly for Shannon’s, as both entropies reach their maximum under the same conditions [17]. The Rényi entropy is calculated using the following formula:

$$R_{en}=\frac{1}{1-p}\log\left[\int_{0}^{\infty }(f(x){)}^{p}\;dx\right],\;p\geq 0,p\neq 1,$$ (7)

where, *p* is a parameter that leads to a positive entropy. The R ényi entropy is known as the quadratic entropy when *p* = 2.

$$R_{en}=\log\left[\frac{(\alpha \beta {)}^{p}}{\beta }\right]+\log\left(\frac{\Gamma \left[\frac{p(\beta -1)+1}{\beta }\right]\Gamma \left[\frac{p(\alpha \beta +1)-1}{\beta }\right]}{\Gamma \left[p(\alpha +1)\right]}\right),$$ (8)

where Γ(·) is the complete gamma function. Eq 8 exists if and only if p(β−1)+1>0, which is always satisfied if β≥1, a condition considered in the subsequent simulation study.

**Havrda and Charvát entropy:** [18] proposed an extension of Rényi’s entropy, known as Havrda and Charvát (HC) entropy, which is defined as:

$$HC=\frac{1}{2^{1-p}-1}\left[\int_{0}^{\infty }(f(x){)}^{p}\;dx-1\right],\;p\geq 0,p\neq 1,$$ (9)

$$HC=\frac{(\alpha \beta {)}^{p}\Gamma \left[\frac{p(\beta -1)+1}{\beta }\right]\Gamma \left[\frac{p(\alpha \beta +1)-1}{\beta }\right]}{\beta \Gamma \left[\alpha p+p\right]}-\frac{1}{2^{1-p}-1}.$$ (10)

*HC* entropy is often used in the context of fuzzy set theory and information retrieval, offering robustness in cases with incomplete or uncertain information.

**Arimoto (A) entropy:** [19] suggested another generalization of Shannon entropy, defined as:

$$A=\frac{p}{1-p}\left\{{\left[\int_{0}^{\infty }(f(x){)}^{p}\;dx\right]}^{1/p}-1\right\},\;p\geq 0,p\neq 1,$$ (11)

$$A=\frac{p}{1-p}\left\{{\left[\frac{(\alpha \beta {)}^{p}\;\Gamma \left(\frac{p(\beta -1)+1}{\beta }\right)\;\Gamma \left(\frac{p(\alpha \beta +1)-1}{\beta }\right)}{\beta \;\Gamma (\alpha p+p)}\right]}^{1/p}-1\right\}$$ (12)

**Tsallis (T) entropy:** [20] generalized Shannon entropy and defined it as:

$$T=\frac{1}{p-1}\left[1-\int_{0}^{\infty }(f(x){)}^{p}\;dx\right],$$ (13)

$$T=\frac{1-(\alpha \beta {)}^{p}\Gamma \left[\frac{p(\beta -1)+1}{\beta }\right]\Gamma \left[\frac{p(\alpha \beta +1)-1}{\beta }\right]}{\left(p-1\right)\beta \Gamma \left[\alpha p+p\right]}.$$ (14)

Several researchers have studied entropy estimates for different life distributions. [11,21] used progressive censoring to investigate entropy in the Burr XII distribution based on ranked set sampling. [22] addressed entropy estimates for the Rayleigh distribution using doubly generalized Type-II hybrid censoring. [23] considered entropy estimators for the inverse Lomax distribution via a multiple censored scheme. [24,25] used non-informative prior to estimate the Shannon entropy of the Lomax distribution. Additionally, [26] evaluated the performance of maximum likelihood and Bayesian models under progressively censored samples. [27] applied Bayesian methods to Shannon entropy for the Burr XII distribution using progressive Type-II censored data. [28,29] evaluated the accuracy of estimators using entropy measures for the Log-Logistic distribution. [30,31] also examined the Shannon entropy of the inverse Weibull distribution under progressive first-failure censoring, comparing credible intervals to asymptotic intervals. [32] used Monte Carlo simulations to illustrate Shannon’s entropy estimates for progressively censored Maxwell distributions. In this study, we explore these entropy measures within the context of the Burr XII distribution, which is known for its versatility in modeling non-monotonic failure rates.

### 1.3 Progressive Type-II censoring scheme

In reliability studies, manufacturers seek to understand the failure time distributions of their products to ensure they meet high-quality standards and have a long lifespan. This understanding is typically gained through life-testing experiments. However, such experiments can be challenging due to time constraints and associated costs, especially when the experiment must be stopped before all items fail. The data obtained from such prematurely ended experiments are known as censored samples. Censoring is a practical technique used in life-testing experiments to save time and money, although it can lead to losing potentially essential data.

In the context of life-testing experiments, two widely recognized approaches are Type-I and Type-II censoring. With Type-I censoring, the experiment is terminated once a specific time has passed. In contrast, Type-II censoring involves ending the experiment only after a certain number of failures. However, these conventional schemes do not allow for the removal of surviving items during the experiment other than at the final termination point.

To address these limitations, progressive Type-II censoring allows for the intermediate removal of surviving units throughout the experiment, providing a more flexible and practical approach. With this type of censoring, *n* independent and identically distributed items are simultaneously placed in a life-testing experiment, and only *m* (<*n*) failures are fully observed. The experiment progresses through *m* stages: after the first failure occurs, a predetermined number of surviving units, *R*_1_, are randomly selected from the remaining *n*–1 units, leaving *n*−1−*R*_1_ surviving items. In the event that the second item fails, the sample becomes *n*−2−*R*_1_, and another sample of size *R*_2_ is randomly selected and removed from the remaining units. This process continues until *m* failures are observed, and all the remaining $n-m-R_{1}-\ldots -R_{m-1}(=R_{m})$ surviving units are removed from the experiment. It is assumed that the lifetimes of these *n* units are independent and identically distributed with a common distribution function *F*(*x*). Further, *n*, *m*, and the censoring scheme $R=(R_{1},R_{2},\ldots ,R_{m})$ are all predetermined. If $R_{1}=R_{2}=\ldots =R_{m-1}=0$, then *R*_*m*_ = *n*−*m*, corresponding to Type-II censoring. If $R_{1}=R_{2}=\ldots =R_{m}=0$, then *m* = *n* represents the complete data set.

This method is particularly useful when units need to be removed from the test due to practical considerations, such as reallocating them for other purposes or observing degradation in a different context. Progressive Type-II censoring, a generalized form of the traditional Type-II censoring method, empowers researchers to adjust it to suit various experimental needs. If all *R*_*i*_ values are set to zero, the scheme reduces to standard Type-II censoring, where the experiment continues until all *m* failures are observed without any intermediate removals.

The flexibility and practicality of progressive Type-II censoring have led to increased interest in this method, especially with the availability of high-speed computing. This technological advancement facilitates extensive simulation studies and more efficient data collection, making researchers feel optimistic and forward-thinking about the future of reliability studies. For those interested in a more detailed discussion of progressive censoring schemes, the works of [32] provide a comprehensive overview.

This is the first study, to our knowledge, to specifically investigate these five entropy estimators within the context of the Burr XII distribution using progressively Type-II censored data. Our findings, including analytical expressions for each entropy measure, maximum likelihood estimators (MLEs), two-sided approximate confidence intervals for all five entropy indices, and numerical comparisons, provide valuable insights into the most effective entropy estimator.

This paper is composed of six sections. Sect 2 discusses the maximum likelihood estimation of Burr XII distribution parameters under progressively Type-II censoring, as well as the derivation of maximum likelihood estimators for the five entropy measures: Shannon, Rényi, Havrda-Charvát, Arimoto, and Tsallis. The delta method is employed in Sect 3 to derive asymptotic confidence intervals for each of the five entropy measures. Sect 4 comprehensively compares the entropy estimators through a simulation study, analyzing their performance in bias, variance, coverage probability, and confidence interval length under different censoring schemes. Sect 5 applies the proposed methods to real-life data from the Wisconsin Breast Cancer Database (WBCD), using the perimeter-worst biomarker to assess the uncertainty between benign and malignant patient groups. Finally, Sect 6 concludes the paper by summarizing the key findings and emphasizing the practical applicability of the entropy measures.

## 2 Maximum likelihood estimation

The maximum likelihood estimation (MLE) method is often regarded as one of the most potent and acceptable approaches for drawing statistical inferences due to its consistency, sufficiency, invariance, and asymptotic efficiency. While MLE can be computationally intensive in some cases, advances in computational power and software have made it more accessible and feasible for complex models and large data sets.

In this section, we focus on the estimation of the parameters of the Burr XII distribution under progressive Type-II censoring scheme. We begin by deriving the MLEs for the shape parameters α and β, which are essential for the subsequent analysis of the entropy measures. These estimators form the foundation for calculating the entropy measures and constructing their associated confidence intervals, ensuring that the characteristics of the censoring scheme is accurately reflected in the results.

### 2.1 Model description

Suppose that *n* independent units are placed in a life-testing experiment whose lifetimes have Burr XII distribution with parameters α and β, with the pdf and cdf as shown in Eqs (1) and (2). The corresponding number of units removed from the test is denoted $\left(R_{1},...,R_{m}\right)$. Let $X_{1:m:n}\leq X_{2:m:n}\leq \cdots \leq X_{m:m:n}$ denote the above mentioned *m* progressively Type-II censoring. For simplicity of notation, we will write *X*_*i*_ to represent *X*_*i*:*m*:*n*_. The likelihood function based on progressively type-II censored sample (see Balakrishnan and Cramer (2014)) is given by:

$$L\propto \overset{m}{\underset{i=1}{\Pi }}f(x_{i};\alpha ,\beta )[1-F(x_{i};\alpha ,\beta ){]}^{R_{i}}$$ (15)

It is usually easier to maximize the logarithm of the likelihood function rather than the likelihood function. Therefore, the log-likelihood function is given by:

$$\ell_{p}(X;\alpha ,\beta )\propto m\log(\alpha \beta )+\left(\beta -1\right)\underset{i=1}{\overset{m}{\sum }}\log\left(x_{i}\right)-\overset{m}{\underset{i=1}{\sum }}(\alpha (R_{i}+1)+1)\log(1+x_{i}^{\beta }).$$ (16)

The *MLEs* of the parameters α and β can be obtained by deriving the likelihood function (16) with respect to α and β and equating the normal equations to 0 as follows:

$$\frac{\partial \ell_{p}(X;\alpha ,\beta )}{\partial \alpha }=\frac{m}{\alpha }-\overset{m}{\underset{i=1}{\sum }}(R_{i}+1)\log(1+x_{i}^{\beta })=0,$$ (17)

$$\frac{\partial \ell_{p}(X;\alpha ,\beta )}{\partial \beta }=\frac{m}{\beta }+\underset{i=1}{\overset{m}{\sum }}\log\left(x_{i}\right)-\overset{m}{\underset{i=1}{\sum }}\frac{\log\left(x_{i}\right)(\alpha (R_{i}+1)+1)x_{i}^{\beta }}{\left(1+x_{i}^{\beta }\right)}=0,$$ (18)

note that from Eqs (17) and (18) we arrive at an optimization problem which is one dimensional. In fact, from (17) we have

$$\alpha =\alpha (\beta )=\frac{m}{\overset{m}{\underset{i=1}{\sum }}(R_{i}+1)\log(1+x_{i}^{\beta })},$$ (19)

when (19) is plugged in (18), the equation will be reduced to a one dimensional nonlinear normal equation of β

$$\frac{m}{\beta }=-\underset{i=1}{\overset{m}{\sum }}\log\left(x_{i}\right)+\overset{m}{\underset{i=1}{\sum }}\frac{\log\left(x_{i}\right)(\alpha (\beta )(R_{i}+1)+1)x_{i}^{\beta }}{\left(1+x_{i}^{\beta }\right)}.$$ (20)

We use Newton Raphson algorithm to solve for the *MLE* of $\beta \left(\hat{\beta }\right)$. Inserting this value into (19), we obtain the *MLE* of $\alpha \left(\hat{\alpha }\right)$.

Now that α and β have been estimated, we utilize the invariance property to obtain the MLEs for the entropy measures. Consequently, the MLE estimator of *S*, denoted by $\hat{S}$ is obtained by inserting $\hat{\alpha }$ and $\hat{\beta }$ in 6 as follows:

$$\hat{S}=-(\frac{1}{\hat{\beta }}-1)(\psi (\hat{\alpha })+\gamma )+\frac{1}{\hat{\alpha }}-\log(\hat{\alpha }\hat{\beta })+1,$$ (21)

similarly for the other entropy estimators, denoted by $\hat{R_{e}n},\hat{HC},\hat{A}$ and $\hat{T}$ are obtained in a similar fashion and given below

$$\hat{R_{en}}=\log\left[\frac{(\hat{\alpha }\hat{\beta }{)}^{p}}{\hat{\beta }}\right]+\log\left(\frac{\Gamma \left[\frac{p(\hat{\beta }-1)+1}{\hat{\beta }}\right]\Gamma \left[\frac{p(\hat{\alpha }\hat{\beta }+1)-1}{\hat{\beta }}\right]}{\Gamma \left[p(\hat{\alpha }+1)\right]}\right),$$ (22)

$$\hat{HC}=\frac{(\hat{\alpha }\hat{\beta }{)}^{p}\Gamma \left[\frac{p(\hat{\beta }-1)+1}{\hat{\beta }}\right]\Gamma \left[\frac{p(\hat{\alpha }\hat{\beta }+1)-1}{\hat{\beta }}\right]}{\hat{\beta }\Gamma \left[\hat{\alpha }p+p\right]}-\frac{1}{2^{1-p}-1},$$ (23)

$$\hat{A}=\frac{p}{1-p}\left\{{\left[\frac{(\hat{\alpha }\hat{\beta }{)}^{p}\Gamma \left[\frac{p(\hat{\beta }-1)+1}{\hat{\beta }}\right]\Gamma \left[\frac{p(\hat{\alpha }\hat{\beta }+1)-1}{\hat{\beta }}\right]}{\beta \Gamma \left[\hat{\alpha }p+p\right]}\right]}^{1/p}-1\right\},$$ (24)

$$\hat{T}=\frac{1-(\hat{\alpha }\hat{\beta }{)}^{p}\Gamma \left[\frac{p(\hat{\beta }-1)+1}{\hat{\beta }}\right]\Gamma \left[\frac{p(\hat{\alpha }\hat{\beta }+1)-1}{\hat{\beta }}\right]}{\left(p-1\right)\hat{\beta }\Gamma \left[\hat{\alpha }p+p\right]}.$$ (25)

## 3 Confidence intervals for entropy measures

The asymptotic confidence intervals (*CIs*) for entropy measures are derived to quantify the uncertainty of the estimates obtained from the Burr XII distribution under progressive Type-II censoring. These intervals are based on the normal approximation of the maximum likelihood estimators.

To construct these confidence intervals, we apply the delta method. This approach, combined with the observed Fisher information matrix, allows us to estimate the variance and covariance of the entropy measures, providing the necessary components for the *CI* calculation. For a detailed explanation of the delta method and its applications, see [33].

The asymptotic 100(1−δ)% confidence interval for each entropy measure are obtained by:


$$\hat{E}\pm Z_{\delta /2}\sqrt{\hat{Var}(\hat{E})},$$

where, $\hat{E}$ is the estimated entropy measure, $Z_{\delta /2}$ denotes the upper (δ/2)th percentile of the standard normal distribution, and $\hat{Var}(\hat{E})$ represents the estimated variance of $\hat{E}$ derived through the delta method.

### 3.1 Delta method and fisher information matrix

The variance of each entropy estimator is computed using the delta method, which approximates the variance as:


$$\hat{Var}(\hat{E})\approx \xi_{E}^{T}{𝐈}^{-1}(\hat{\alpha },\hat{\beta })\xi_{E}$$

where $\xi_{E}={\left(\frac{\partial E}{\partial \alpha },\frac{\partial E}{\partial \beta }\right)}^{⊤}$ evaluated at $\left(\alpha ,\beta )=(\hat{\alpha },\hat{\beta }\right)$, and ${𝐈}^{-1}(\hat{\alpha },\hat{\beta })$ is the inverse of the observed Fisher information matrix at the estimated parameters $\hat{\alpha }$ and $\hat{\beta }$. For the computation of the Fisher information matrix, the second derivatives of the log-likelihood function with respect to the parameters α and β are required. These derivatives have been obtained using Mathematica 13 and are detailed in the appendix (see Appendix A).

### 3.2 Confidence intervals for specific entropy measures

1. **Shannon Entropy (S):** The variance of the Shannon entropy estimator, $\hat{Var}(\hat{S})$, is derived from the Fisher information matrix and partial derivatives of the Burr XII distribution. The confidence interval is then given by:
  $$\hat{S}\pm Z_{\delta /2}\sqrt{\hat{Var}(\hat{S})}.$$ (26)
2. **Rényi Entropy (*R***_***en***_**):** The confidence interval for R ényi entropy follows a similar approach. The variance, $\hat{Var}({\hat{R}}_{en})$, is computed using the corresponding partial derivatives and Fisher information matrix. The confidence interval is:
  $${\hat{R}}_{en}\pm Z_{\delta /2}\sqrt{\hat{Var}({\hat{R}}_{en})}$$ (27)
3. **Havrda-Charvát Entropy (HC):** For Havrda-Charvát entropy, the variance $\hat{Var}(\hat{HC})$ is obtained similarly, and the confidence interval is:
  $$\hat{HC}\pm Z_{\delta /2}\sqrt{\hat{Var}(\hat{HC})}$$ (28)
4. **Arimoto Entropy (A):** The Arimoto entropy variance $\hat{Var}(\hat{A})$ is derived, leading to the confidence interval:
  $$\hat{A}\pm Z_{\delta /2}\sqrt{\hat{Var}(\hat{A})}$$ (29)
5. **Tsallis Entropy (T):** Finally, for Tsallis entropy, the variance $\hat{Var}(\hat{T})$ and the confidence interval are computed as:
  $$\hat{T}\pm Z_{\delta /2}\sqrt{\hat{Var}(\hat{T})}$$ (30)

These confidence intervals provide a rigorous means to quantify the uncertainty associated with the entropy estimators under the progressively Type-II censoring scheme. For detailed mathematical derivations and the specific forms of the second derivatives used in the Fisher information matrix, refer to Appendix A.

## 4 Simulation study

This simulation study is designed to rigorously evaluate the performance of maximum likelihood estimators for the five proposed entropy measures. These estimators are derived from various sets of progressive Type-II censored samples generated from the Burr XII distribution, following the methodology described by [32], $x_{1},x_{2},...,x_{m}$ is the progressive Type-II censored sample of size *m* from the Burr XII distribution with parameters α and β.

The maximum likelihood estimates of the parameters *α* and *β* are obtained using Eqs 19 and 20. These estimates are then employed to calculate the five entropy measures: Rényi, Shannon, Havrda-Charvát, Arimoto, and Tsallis. To assess the performance of these estimators, we evaluate their absolute bias (Bias), asymptotic variance (Var), and the properties of their 95 The simulation study consists of 1000 replications. For the Burr XII distribution, two sets of parameter values, (α,β)=(2,2) and (5,2), are considered. Three censoring schemes (Cs) are used in the simulation:

- **Scheme 1 (Sc1):** $R_{2}=R_{3}=\cdots =R_{m}=1$, and *R*_1_ = *n*−2*m* + 1,
- **Scheme 2 (Sc2):** $R_{2}=R_{3}=\cdots =R_{m}=2$, and *R*_1_ = *n*−3*m* + 2,
- **Scheme 3 (Sc3):** $R_{2}=R_{3}=\cdots =R_{m}=0$, and *R*_1_ = *n*−*m*.

The sample size used is *n* = 100, with effective sample size m=20,40,60. The analysis is conducted for four values of the parameter *p*, specifically p=0.5,0.7,1.5,3.

Results of the simulation are summarized in Tables 1–4. Due to space limitations, only some of the simulation results are presented. The remaining results exhibit similar patterns.

**Table 1:** Absolute bias (Bias), asymptotic variance (Var), asymptotic length (L), and coverage probability (Cov) when *p* = 0.5 and for *n* = 100, α=5,β=2 and for different values of m.

m		Scheme 1					Scheme 2					Scheme 3				
${\hat{R}}_{en}$	$\hat{HC}$	$\hat{A}$	$\hat{T}$	$\hat{S}$	${\hat{R}}_{en}$	$\hat{HC}$	$\hat{A}$	$\hat{T}$	$\hat{S}$	${\hat{R}}_{en}$	$\hat{HC}$	$\hat{A}$	$\hat{T}$	$\hat{S}$
20	Bias	.054	.046	.019	.094	.054	.063	.050	.015	.012	.065	.041	.037	.020	.016	.041
	Var	.075	.142	.137	.097	.054	.102	.201	.208	.138	.062	.046	.084	.076	.058	.029
	L	.659	.900	.669	.782	.300	.736	1.018	.762	.885	.358	.452	.601	.551	.639	.179
	Cov	.912	.891	.867	.983	.914	.909	.886	.858	.990	.916	.907	.895	.878	.907	.910
40	Bias	.032	.028	.017	.013	.032	.039	.033	.018	.011	.038	.022	.021	.014	.015	.022
	Var	.039	.070	.063	.048	.023	.053	.099	.092	.068	.032	.023	.041	.036	.028	.014
	L	.531	.715	.534	.619	.206	.592	.804	.605	.698	.252	.364	.483	.441	.501	.117
	Cov	.924	.905	.889	.984	.925	.922	.902	.888	.997	.927	.924	.908	.898	.924	.918
60	Bias	.028	.028	.013	.012	.027	.034	.032	.012	.010	.033	.020	.020	.012	.008	.018
	Var	.026	.047	.040	.032	.016	.036	.065	.058	.045	.022	.015	.027	.023	.019	.009
	L	.374	.496	.453	.539	.121	.424	.563	.516	.607	.154	.306	.405	.368	.432	.081
	Cov	.925	.914	.904	.989	.930	.923	.907	.889	.998	.928	.934	.927	.918	.924	.939

**Table 2:** Absolute bias (Bias), asymptotic variance (Var), asymptotic length (L), and coverage probability (Cov) when p=0.7 and for n=100, α=5,β=2 and for different values of *m.*

m		Scheme 1					Scheme 2					Scheme 3				
		${\hat{R}}_{en}$	$\hat{HC}$	$\hat{A}$	$\hat{T}$	$\hat{S}$	${\hat{R}}_{en}$	$\hat{HC}$	$\hat{A}$	$\hat{T}$	$\hat{S}$	${\hat{R}}_{en}$	$\hat{HC}$	$\hat{A}$	$\hat{T}$	$\hat{S}$
20	Bias	.054	.072	.051	.290	.054	.064	.069	.048	.059	.065	.041	.046	.033	.0398	.0412
	Var	.056	.099	.060	.059	.045	.076	.135	.083	.080	.062	.035	.061	.037	.0362	.0286
	L	.382	.508	.396	.519	.236	.435	.579	.452	.610	.283	.316	.419	.326	.391	.1787
	Cov	.915	.905	.898	.766	.914	.914	.900	.895	.854	.916	.907	.902	.897	.579	.910
40	Bias	.032	.035	.025	.045	.032	.039	.042	.030	.047	.038	.022	.025	.018	.039	.022
	Var	.029	.051	.031	.030	.023	.040	.070	.043	.042	.032	.017	.030	.018	.018	.014
	L	.300	.399	.310	.387	.164	.342	.455	.354	.458	.201	.247	.327	.254	.286	.117
	Cov	.925	.918	.910	.800	.925	.924	.912	.908	.867	.926	.924	.921	.912	.637	.918
60	Bias	.027	.032	.023	.041	.027	.033	.038	.028	.042	.033	.019	.022	.016	.039	.018
	Var	.019	.034	.021	.020	.016	.027	.047	.029	.028	.022	.012	.020	.012	.012	.009
	L	.252	.333	.259	.325	.121	.289	.384	.299	.386	.154	.204	.270	.210	.238	.081
	Cov	.929	.923	.922	.809	.930	.925	.916	.912	.871	.927	.936	.931	.931	.683	.939

**Table 3:** Absolute bias (Bias), asymptotic variance (Var), asymptotic length (L), and coverage probability (Cov) when p=1.5 and for n=100,α=5,β=2 and for different values of *m.*

m		Scheme 1					Scheme 2					Scheme 3				
		${\hat{R}}_{en}$	$\hat{HC}$	$\hat{A}$	$\hat{T}$	$\hat{S}$	${\hat{R}}_{en}$	$\hat{HC}$	$\hat{A}$	$\hat{T}$	$\hat{S}$	${\hat{R}}_{en}$	$\hat{HC}$	$\hat{A}$	$\hat{T}$	$\hat{S}$
20	Bias	.079	.162	.0892	.3013	.1314	.100	.206	.113	.362	.159	.056	.115	.064	.219	.098
	Var	.039	.141	.0447	.0484	.0452	.052	.193	.061	.066	.061	.025	.089	.028	.030	.029
	L	.489	.947	.5315	.7112	.4672	.552	1.077	.603	.826	.550	.414	.791	.446	.566	.375
	Cov	.925	.942	.940	.716	.925	.930	.943	.939	.716	.928	.922	.938	.929	.712	.920
40	Bias	.038	.079	.043	.190	.079	.052	.107	.059	.230	.101	.023	.049	.027	.135	.054
	Var	.020	.070	.023	.024	.023	.027	.098	.031	.034	.032	.012	.043	.014	.015	.014
	L	.358	.679	.384	.511	.319	.404	.773	.436	.596	.378	.295	.556	.315	.403	.248
	Cov	.941	.948	.947	.721	.939	.937	.948	.947	.723	.936	.945	.954	.950	.713	.939
60	Bias	.027	.056	.031	.141	.062	.037	.076	.042	.172	.080	.017	.035	.019	.100	.041
	Var	.013	.047	.015	.016	.016	.019	.066	.021	.023	.022	.008	.028	.009	.010	.009
	L	.314	.591	.335	.422	.270	.357	.676	.383	.495	.319	.257	.482	.274	.332	.213
	Cov	.954	.964	.961	.716	.950	.952	.956	.956	.724	.949	.954	.964	.961	.720	.950

**Table 4:** Absolute bias (Bias), asymptotic variance (Var), asymptotic length (L), and coverage probability (Cov) when p=3 and for n=100,α=5,β=2 and for different values of *m.*

m		Scheme 1					Scheme 2					Scheme 3				
		${\hat{R}}_{en}$	$\hat{HC}$	$\hat{A}$	$\hat{T}$	$\hat{S}$	${\hat{R}}_{en}$	$\hat{HC}$	$\hat{A}$	$\hat{T}$	$\hat{S}$	${\hat{R}}_{en}$	$\hat{HC}$	$\hat{A}$	$\hat{T}$	$\hat{S}$
20	Bias	0.060	0.518	0.089	0.287	0.131	0.073	0.664	0.111	0.376	0.159	0.045	0.363	0.066	0.188	0.098
	Var	0.033	1.332	0.055	0.187	0.045	0.045	0.093	0.076	0.283	0.061	0.022	0.731	0.035	0.103	0.029
	L	0.539	3.077	0.689	1.357	0.467	0.609	3.618	0.787	1.605	0.550	0.451	2.462	0.570	1.065	0.375
	Cov	0.930	0.967	0.953	0.902	0.925	0.933	0.957	0.952	0.921	0.928	0.924	0.952	0.935	0.843	0.920
40	Bias	0.029	0.244	0.043	0.142	0.079	0.036	0.322	0.055	0.189	0.101	0.020	0.160	0.030	0.090	0.054
	Var	0.017	0.529	0.027	0.074	0.023	0.024	0.794	0.038	0.112	0.032	0.011	0.299	0.017	0.042	0.014
	L	0.386	2.04	0.483	0.931	0.319	0.444	2.405	0.560	1.095	0.378	0.312	1.604	0.388	0.737	0.248
	Cov	0.934	0.959	0.940	0.846	0.939	0.941	0.962	0.941	0.850	0.936	0.939	0.955	0.945	0.856	0.939
60	Bias	0.022	0.172	0.032	0.099	0.062	0.027	0.223	0.040	0.128	0.080	0.015	0.113	0.022	0.064	0.041
	Var	0.011	0.329	0.018	0.046	0.016	0.016	0.486	0.025	0.068	0.017	0.007	0.189	0.011	0.027	0.007
	L	0.330	0.697	0.410	0.767	0.270	0.384	1.998	0.479	0.902	0.319	0.265	1.336	0.327	0.603	0.213
	Cov	0.943	0.953	0.951	0.845	0.950	0.942	0.998	0.949	0.855	0.949	0.945	0.957	0.948	0.879	0.950

### 4.1 Data analysis and comparison study

It is evident from Tables 1–4 that the performance of five entropy estimators consistently decreases as the effective sample size *m* increases across all schemes, particularly for the Renyi and Havrda-Charvát measures. The Tsallis and Arimoto estimators also demonstrate enhanced Bias reduction with increasing *m*. However, the Tsallis estimator displays higher Bias at smaller *m* and smaller *p* values, rendering it less dependable under those conditions.

The variance (Var) and confidence interval length (L) decrease as *m* increases. The Renyi estimator generally has the smallest variance for different values of *p*, followed closely by Havrda-Charvát and Arimoto. Tsallis shows higher variance, especially at smaller *m* and smaller *p*, but improves as *m* increases. Shannon is unaffected by *p* changes and consistently shows small Bias, variance, and interval length across all schemes.

Regarding coverage probability (Cov), most estimators approach the nominal level of 0.95 as the sample size (*m*) increases. Renyi and Havrda-Charvát consistently achieve coverage close to 0.95 across all scenarios. Meanwhile, Tsallis tends to perform poorly with smaller sample sizes and values of *p*. Overall, the Renyi and Havrda-Charvát estimators offer the best balance between minimal Bias, low variance, and appropriate coverage probability, making them the most reliable across different scenarios.

The analysis results suggest that when choosing an entropy measure for practical use, the sample size and parameter *p* must be considered. Shannon entropy is a strong choice due to its stability. However, Tsallis and Havrdát can also be reliable estimation methods when used under specific conditions (such as larger *m*, lower *p*, and a favorable scheme).

## 5 Real life data analysis

For the diagnosis of breast cancer, we utilized summary features from digitized images of a fine needle aspirate (FNA) of breast masses, which serve as biomarkers. This section applies the proposed entropy measures to assess the uncertainty between benign and malignant patients using data from the Wisconsin Breast Cancer Database (WBCD), created by the University of Wisconsin [34]. The dataset consists of 569 observations and 30 features, with the variable “Diagnosis” serving as the gold standard, where B = benign (n=357) and M = malignant (n=212). Among the 30 features, we selected the perimeter-worst biomarker due to its superior diagnostic performance. This biomarker demonstrates high sensitivity (0.920) and specificity (0.919), with a Youden Index of 0.839, outperforming other biomarkers in differentiating between benign and malignant cases.

The legitimacy of the Burr XII model for both benign and malignant data is assessed based on $\left(\alpha_{1},\beta_{1})=(1.6305,9.4945\right)$ for the benign group and $\left(\alpha_{2},\beta_{2})=(1.2270,11.1030\right)$ for the malignant group, using Kolmogorov-Smirnov (K-S), Anderson-Darling (A-D), and chi-squared tests. The results, presented in Table 5 at a significance level of 0.05, provide strong evidence that the Burr XII model fits both datasets well.

**Table 5:** Test statistic and p-value associated with each test for Benign and Malignant patient groups.

Data	K-S( p-value)	A-D( p-value)	chi-squared( p-value)
Benign	0.0379 (**0.6711**)	0.4960 (**0.4995**)	6.7356 (**0.5654**)
Malignant	0.0381 (**0.9073**)	0.2716 (**0.4731**)	6.6782 (**0.4631**)

**Fig 3:**
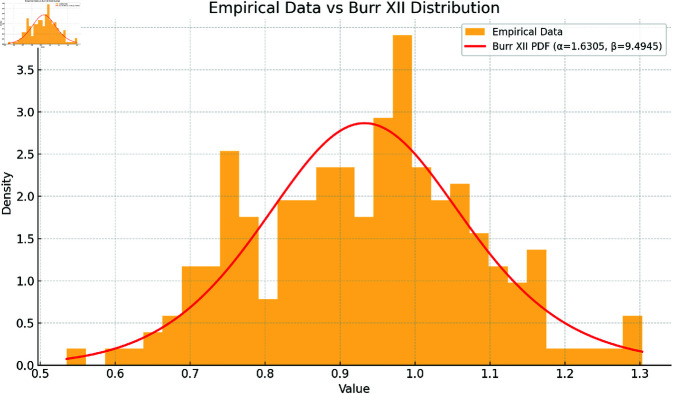
Histogram plot for Benign Group.

**Fig 4:**
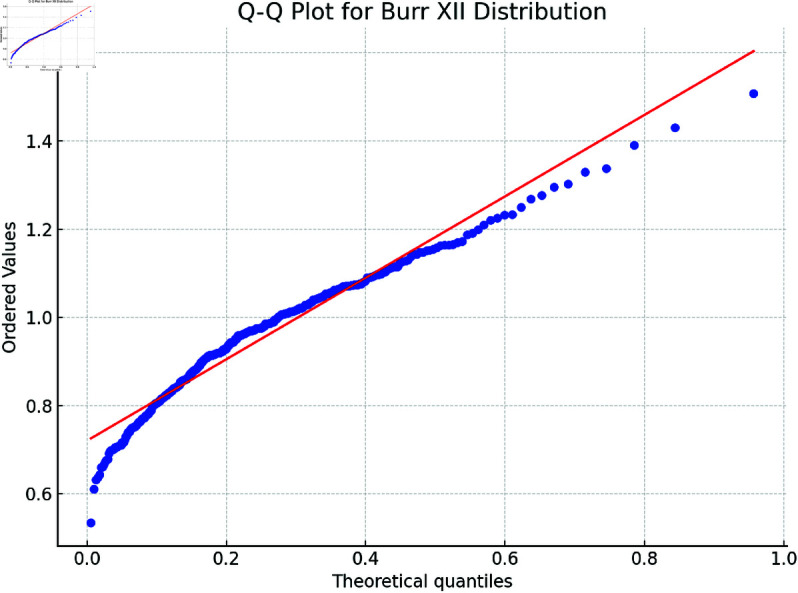
Q-Q Plot for Benign Group.

**Fig 5:**
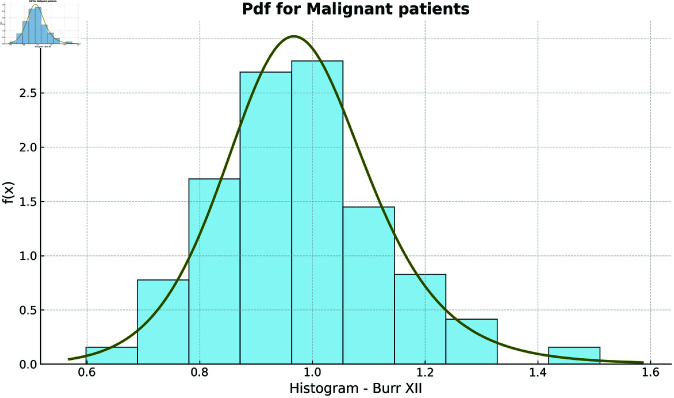
Histogram Plot for Malignant Group.

**Fig 6:**
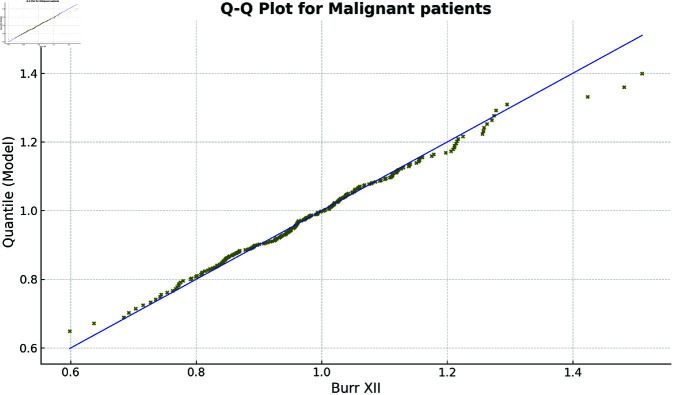
Q-Q Plot for Malignant Group.

Additionally, the fitted pdfs and Q-Q plots for the benign and malignant datasets, shown in Figs 3 and 4 (benign group) and Figs 5 and 6 (malignant group), respectively, further confirm the Burr XII distribution as a suitable model for both datasets.

In this analysis, we apply the three different censoring schemes (Sc) described in Sect 4 to estimate the entropy measures for both the benign and malignant groups. The sample sizes for the entropy estimates were *m*_1_ = 119 for the benign group and *m*_2_ = 90 for the malignant group. We calculated the entropy measures and presented the results, including MLEs, Bias, asymptotic length (L), and asymptotic variance (Var), in Table 6.

**Table 6:** Maximum Likelihood Estimates (MLE), Bias, Asymptotic Length (L), and Variance (Var) of Entropy Measures for Benign and Malignant patients Data based on *p* = 0.3.

Benign Data						Malignant Data				
	$\hat{R_{en}}$	$\hat{HC}$	$\hat{A}$	$\hat{T}$	$\hat{S}$	${\hat{R}}_{en}$	$\hat{HC}$	$\hat{A}$	$\hat{T}$	$\hat{S}$
Exact	0.0473	0.0539	0.0500	0.0481	-0.4740	0.0473	0.0539	0.0500	0.0481	-0.4740
Scheme 1						Scheme 1				
MLE	0.1974	0.2372	0.2506	0.1897	-0.3737	0.1934	0.2322	0.2445	0.1919	-0.3774
Bias	0.1501	0.1833	0.2006	0.1416	0.1002	0.1462	0.1783	0.1945	0.1438	0.0966
Var	0.0112	0.0185	0.0281	0.0148	0.0068	0.0113	0.0185	0.0277	0.0147	0.0068
L	0.3947	0.4744	0.5013	0.3794	0.3232	0.3869	0.4644	0.4890	0.3837	0.3243
Scheme 2						Scheme 2				
MLE	0.4580	0.6052	0.8191	0.4166	-0.2028	0.4944	0.6622	0.930	0.4465	-0.1793
Bias	0.4107	0.5513	0.7691	0.3685	0.2711	0.4471	0.6083	0.8800	0.3984	0.2946
Var	0.0159	0.0380	0.1350	0.0302	0.0075	0.0167	0.0420	0.1680	0.0334	0.0076
L	0.4948	0.7642	1.4405	0.6818	0.3392	0.5068	0.8030	1.6065	0.7164	0.3423
Scheme 3						Scheme 3				
MLE	0.1132	0.1321	0.1296	0.1614	-0.3750	0.1315	0.1539	0.1551	0.1414	-0.3750
Bias	0.0659	0.0782	0.0796	0.2095	0.0990	0.0646	0.0773	0.0824	0.0728	0.0994
Var	0.0057	0.0084	0.0097	0.0067	0.0039	0.0058	0.0088	0.0111	0.0070	0.0038
L	0.2265	0.2642	0.2592	0.3205	0.2443	0.2630	0.3078	0.3103	0.2828	0.2444

Table 6 reveals that Sc1 and Sc3 produce more precise estimates for all entropy measures, particularly in the benign group, where lower Bias and variance are observed compared to Sc2. This pattern holds across benign and malignant datasets, with the differences in Bias and variance being more pronounced in the malignant group, where Sc2 again produces less reliable estimates.

Shannon entropy presents a notable limitation in this analysis, as it produced negative MLE values for both the benign and malignant datasets. This complicates its interpretation, particularly for continuous distributions like the Burr XII, and raises questions about its suitability for this context. The negative values may indicate potential misalignment between the data and the assumed distribution model, making Shannon entropy less reliable for measuring uncertainty in this dataset.

Among the entropy measures examined, Rényi entropy emerged as the most reliable indicator of uncertainty, consistently producing positive values across all schemes. This contrasts with Shannon entropy, which yielded negative MLE values, and the Arimoto entropy, which exhibited higher Bias and variance under Sc2. Rényi entropy’s consistent performance across schemes and stability in both benign and malignant groups suggests its robustness in distinguishing between these two populations. Scheme 3 generally provided the most precise estimates, whereas Sc2 produced the least reliable outcomes across all measures.

The perimeter-worst biomarker demonstrated lower uncertainty in the benign group compared to the malignant group across all entropy measures, highlighting its predictive power in distinguishing between diseased (malignant) and non-diseased (benign) cases. This observation supports the effectiveness of the perimeter-worst biomarker as a diagnostic tool for breast cancer, particularly when paired with robust entropy measures like Rényi, which offer greater reliability in quantifying uncertainty in this context.

## 6 Concluding remarks

This study comprehensively evaluated five entropy measures, Shannon, Rényi, Havrda-Charvát, Arimoto, and Tsallis, under progressive Type-II censoring schemes for the Burr XII distribution. The simulation results indicated that Rényi and Havrda-Charvát consistently outperformed the other measures in terms of Bias, variance, and coverage probability, particularly as sample sizes increased. Tsallis and Arimoto improved with larger sample sizes but exhibited higher Bias and variance at smaller sample sizes.

Overall, the simulation results and the real-life example both suggest that Rényi entropy is the most robust and reliable measure of uncertainty, especially for larger sample sizes and across different censoring schemes. Shannon entropy’s stability makes it a strong choice, but its performance in terms of Bias and variance does not surpass that of Rényi or Havrda-Charvát. For practical applications, the choice of entropy estimator should consider both the sample size and the value of *p*, with Rényi and Havrda-Charvát offering the best overall balance between Bias, variance, and coverage probability.

## Appendix A

The entries for approximate confidence intervals are given by the following equations

$$I^{-1}\left(\hat{\alpha },\hat{\beta }\right)={\left[\begin{array}{cc} -\frac{\partial^{2}\ln L(\alpha ,\beta )}{\partial \alpha^{2}} & -\frac{\partial^{2}\ln L(\alpha ,\beta )}{\partial \alpha \partial \beta }\\ -\frac{\partial^{2}\ln L(\alpha ,\beta )}{\partial \beta \partial \alpha } & -\frac{\partial^{2}\ln L(\alpha ,\beta )}{\partial \beta^{2}} \end{array}\right]}_{\left(\alpha ,\beta \right)=\left(\hat{\alpha },\hat{\beta }\right)}^{-1},$$ (31)

where, $I\left(\hat{\alpha },\beta \right)$ is the observed information matrix and

$$\frac{\partial^{2}\ln L(\alpha ,\lambda )}{\partial \alpha^{2}}=-\frac{m}{\alpha^{2}},$$ (32)

$$\frac{\partial^{2}\ln L(\alpha ,\lambda )}{\partial \beta^{2}}=-\frac{m}{\beta^{2}}-\sum_{i=1}^{m}\left(\frac{{\left(\log x_{i}\right)}^{2}\left(1+\alpha +\alpha R_{i}\right)x_{i}^{\beta }}{{\left(1+x_{i}^{\beta }\right)}^{2}}\right),$$ (33)

$$\frac{\partial^{2}\ln L(\alpha ,\lambda )}{\partial \alpha \partial \beta }=-\sum_{i=t1}^{m}\frac{x_{i}^{\beta }\log x_{i}}{x_{i}^{\beta }+1}-\sum_{i=t1}^{m}R_{i}\frac{x_{i}^{\beta }\log x_{i}}{x_{i}^{\beta }+1}-\frac{x_{m}^{\beta }\log x_{m}R^{*}}{1+x_{m}^{\beta }}.$$ (34)

**Shannon entropy**

$\xi_{S}=\left(\frac{\partial S}{\partial \alpha },\frac{\partial S}{\partial \beta }\right){|}_{\left(\alpha ,\beta \right)=\left(\hat{\alpha },\hat{\beta }\right)}$,    $\frac{\partial S}{\partial \alpha }=\frac{-\left(\alpha +1\right)}{\alpha^{2}}+\frac{\left(\beta -1\right)\;\psi^{\prime }\left(\alpha \right)}{\beta },\;\;\frac{\partial S}{\partial \beta }=\frac{S_{0}}{\beta^{2}}$

where $\psi \left(.\right)=\frac{\Gamma^{\prime }\left(.\right)}{\Gamma \left(.\right)}$ is the digamma function and $\Gamma^{\prime }\left(.\right)$ is the first derivative of Γ(.), $\psi^{\prime }\left(\alpha \right)$ is the derivative of the digamma.

$$\hat{Var(\hat{S})}=c_{S}\left[\frac{-c_{S}\;[\frac{m}{\beta^{2}}+total_{1}]-\frac{S_{0}\;total_{2}}{{\hat{\beta }}^{2}}}{\left[\frac{m}{\alpha^{2}}\left(\frac{m}{\beta^{2}}+total_{1}\right)+{\left(total_{2}\right)}^{2}\right]}\right]+\;\frac{S_{0}}{{\hat{\beta }}^{2}}\times \left[\frac{\frac{m\;S_{0}}{\alpha^{2}{\hat{\beta }}^{2}}-c_{S}\;total_{2}}{\left[\frac{m}{\alpha^{2}}\left(\frac{m}{\beta^{2}}+total_{1}\right)+{\left(total_{2}\right)}^{2}\right]}\right],$$ (35)

where,

$c_{S}=\left[-\frac{\left(\alpha +1\right)}{\alpha^{2}}+\frac{\left(\beta -1\right)\psi^{\prime }(\hat{\alpha })}{\beta }\right],\;\;S_{0}=\gamma -\hat{\beta }+\psi (\hat{\alpha }),$

$total1=\frac{m}{{\hat{\beta }}^{2}}+\left(1+\hat{\alpha }\right)\sum_{i=1}^{m}\frac{x_{i}^{\hat{\beta }}{\left[\log x_{i}\right]}^{2}}{(x_{i}^{\hat{\beta }}+1{)}^{2}}+\alpha \sum_{i=1}^{j}R_{i}\frac{x_{i}^{\hat{\beta }}{\left[\log x_{i}\right]}^{2}}{(x_{i}^{\hat{\beta }}+1{)}^{2}}+\frac{x_{m}^{\beta }\;{\left[\log x_{m}\right]}^{2}\;R^{*}}{{\left(1+x_{m}^{\beta }\right)}^{2}},$

$total_{2}=\sum_{i=t1}^{m}\frac{x_{i}^{\hat{\beta }}\log x_{i}}{x_{i}^{\hat{\beta }}+1}+\sum_{i=t1}^{m}R_{i}\frac{x_{i}^{\hat{\beta }}\log x_{i}}{x_{i}^{\hat{\beta }}+1}+\frac{x_{m}^{\beta }\;\log x_{m}\;R^{*}}{\left(1+x_{m}^{\beta }\right)}.$

**Rényi entropy**

$\xi_{R}=\left(\frac{\partial R}{\partial \alpha },\frac{\partial R}{\partial \beta }\right){|}_{\left(\alpha ,\beta \right)=\left(\hat{\alpha },\hat{\beta }\right)}$        $\frac{\partial R}{\partial \alpha }=\frac{p}{\left(p-1\right)\alpha }R_{00}$        $\frac{\partial R}{\partial \beta }=-\frac{R_{0}}{\beta^{2}},$

$$\hat{Var(\hat{R})}=\frac{\hat{\alpha }(1-p)R_{0}\times [-m(p-1)R_{0}+p\hat{\alpha }\beta^{2}(-R_{00})\times Total_{2}]}{\left(\hat{\beta }-\hat{\beta }p{)}^{2}[-{\hat{\alpha }}^{3}{\hat{\beta }}^{2}\times Total_{2}^{2}\;+\;m\;\hat{\alpha }\;S_{1}\right]}\;\;-\frac{p\;\hat{\alpha }{\hat{\text{𝛽}}}^{2}(R_{00})\$}{[}-(p-1)\;\hat{\alpha }\;R_{0}\times total_{2}\;+p(-R_{00})S_{1}]\;\}\left(\hat{\beta }-\hat{\beta }p{)}^{2}[-{\hat{\alpha }}^{3}{\hat{\beta }}^{2}\times Total_{2}^{2}\;+\;m\;\hat{\alpha }\;S_{1}\right],$$ (36)

where,

$R_{0}=\beta +\psi \left(\frac{1+p\left(\beta -1\right)}{\beta }\right)-\psi \left(\frac{p\left(\alpha +\beta +1\right)-1}{\beta }\right)$, $\;R_{00}=-1+\alpha \left[\psi \left(p+p\alpha \right)-\psi \left(\frac{p\left(1+\alpha \beta \right)-1}{\beta }\right)\right]$,

$S_{00}=(1+\hat{\alpha })\hat{\beta }-{\hat{\alpha }}^{2}(\beta -1)$, $\;S_{1}=m+{\hat{\beta }}^{2}\;total_{1},$

$total1=\frac{m}{{\hat{\beta }}^{2}}+\left(1+\hat{\alpha }\right)\sum_{i=1}^{m}\frac{x_{i}^{\hat{\beta }}{\left[\log x_{i}\right]}^{2}}{(x_{i}^{\hat{\beta }}+1{)}^{2}}+\alpha \sum_{i=1}^{j}R_{i}\frac{x_{i}^{\hat{\beta }}{\left[\log x_{i}\right]}^{2}}{(x_{i}^{\hat{\beta }}+1{)}^{2}}+\frac{x_{m}^{\beta }\;{\left[\log x_{m}\right]}^{2}\;R^{*}}{{\left(1+x_{m}^{\beta }\right)}^{2}},$

$total_{2}=\sum_{i=t1}^{m}\frac{x_{i}^{\hat{\beta }}\log x_{i}}{x_{i}^{\hat{\beta }}+1}+\sum_{i=t1}^{m}R_{i}\frac{x_{i}^{\hat{\beta }}\log x_{i}}{x_{i}^{\hat{\beta }}+1}+\frac{x_{m}^{\beta }\;\log x_{m}\;R^{*}}{\left(1+x_{m}^{\beta }\right)}.$

**Havrda and Charvát entropy**

$\xi_{HC}=\left(\frac{\partial HC}{\partial \alpha },\frac{\partial HC}{\partial \beta }\right){|}_{\left(\alpha ,\beta \right)=\left(\hat{\alpha },\hat{\beta }\right)}$     $\frac{\partial HC}{\partial \alpha }=\frac{2^{p}\;p\;{\left(\alpha \beta \right)}^{p-1}\;G_{1}\;G_{2}\;R_{00}}{\left(2^{p}-2\right)\;G_{0}}\;\frac{\partial HC}{\partial \beta }=\frac{2^{p}\;\left(p-1\right)\;{\left(\alpha \beta \right)}^{p}\;G_{1}\;G_{2}\;R_{0}}{\left(2^{p}-2\right)\;\beta^{3}\;G_{0}}$


$$part_{1}=\;(1+2p(p-1)){\hat{\beta }}^{2}+[\hat{\alpha }\;p\;\hat{\beta }\;HN_{\left(p(\hat{\alpha }+1)-1\right)}{]}^{2}+[(p-1)HN_{\left(\frac{\left(p-1)(\hat{\beta }-1\right)}{\hat{\beta }}\right)}{]}^{2}\;+2\hat{\beta }[-1+p(2+p(\hat{\alpha }\hat{\beta }-1))][\gamma +D_{1}]\;+[1+p(-2+p((\hat{\alpha }\hat{\beta }{)}^{2}+1))][\gamma +D_{1}{]}^{2}\;-2(p-1{)}^{2}HN_{\left(\frac{\left(p-1)(\hat{\beta }-1\right)}{\hat{\beta }}\right)}[\gamma -\hat{\beta }+D_{1}]\;-2p^{2}\hat{\alpha }\beta^{2}HN_{\left(p(\hat{\alpha }+1)-1\right)}[1+\hat{\alpha }[\gamma +D_{1}]]\;$$

$$part_{2}=p^{2}\;{\hat{\beta }}^{4}\;R_{00}^{2}\;total_{1}+2\;(p-1)\;p\;\hat{\alpha }\;{\hat{\beta }}^{2}\;R_{0}\;R_{00}\;total_{2}$$

$$Var(HC)=\frac{4^{p}(\hat{\alpha }\hat{\beta }{)}^{2p}(G_{1}G_{2}{)}^{2}[m\;part_{1}+part_{2}]}{\left(2^{p}-2{)}^{2}\;{\hat{\beta }}^{4}\;G_{0}^{2}\;(m^{2}+{\hat{\beta }}^{2}(m\;total_{1}-{\hat{\alpha }}^{2}\;total_{2}^{2})\right)}$$ (37)

where, *HN*_(.)_ is the harmonic-number, γ is Eulergamma. And $D_{0}=\psi (\alpha p+p),$ $D_{1}=\psi (\frac{p(\hat{\alpha }\hat{\beta }+1)-1}{\hat{\beta }}),$ $D_{2}=\psi (\frac{p(\hat{\beta }-1)+1}{\hat{\beta }})$, $G_{0}=\Gamma (\hat{\alpha }p+p),$ $G_{1}=\Gamma (\frac{p(\hat{\alpha }\hat{\beta }+1)-1}{\hat{\beta }}),$ $G_{2}=\Gamma (\frac{p(\hat{\beta }-1)+1}{\hat{\beta }})$, $G_{3}=\Gamma (\frac{\left(p-1)(\hat{\beta }-1\right)}{\hat{\beta }}),$

**Arimoto entropy**

$\xi_{A}{|}_{\left(\alpha ,\beta \right)=\left(\hat{\alpha },\hat{\beta }\right)}=\left(\frac{\partial A}{\partial \alpha },\frac{\partial A}{\partial \beta }\right){|}_{\left(\alpha ,\beta \right)=\left(\hat{\alpha },\hat{\beta }\right)},$ $\frac{\partial A}{\partial \alpha }=\frac{p\;R_{00}}{\alpha \;\left(p-1\right)}{\left[\frac{{\left(\alpha \beta \right)}^{p}\;G_{1}\;G_{2}}{\beta \;G_{0}}\right]}^{\frac{1}{p}},$ $\frac{\partial A}{\partial \beta }=-\frac{R_{0}}{\beta^{2}}{\left[\frac{{\left(\alpha \beta \right)}^{p}\;G_{1}\;G_{2}}{\beta \;G_{0}}\right]}^{\frac{1}{p}}.$

**Tsallis entropy**

$\xi_{T}{|}_{\left(\alpha ,\beta \right)=\left(\hat{\alpha },\hat{\beta }\right)}=\left(\frac{\partial T}{\partial \alpha },\frac{\partial T}{\partial \beta }\right){|}_{\left(\alpha ,\beta \right)=\left(\hat{\alpha },\hat{\beta }\right)},$ $\frac{\partial T}{\partial \alpha }=\frac{p\;\alpha \;\left(\beta -1\right)\;{\left(\alpha \beta \right)}^{p-2}\;G_{1}\;G_{4}\;R_{00}}{G_{0}},$ $\frac{\partial T}{\partial \beta }=\frac{{\left(\alpha \beta \right)}^{p}\;G_{1}\;G_{2}\;R_{0}}{\beta^{3}\;G_{0}},$

$$\hat{Var(\hat{T})}=[\frac{(\hat{\alpha }\hat{\beta }{)}^{p}\;G_{1}\;G_{2}}{\hat{\beta }\;G_{0}}{]}^{\frac{2}{p}}\;\times \frac{\left[m\;part_{1}+part_{2}\right]}{\left(p-1{)}^{2}{\hat{\beta }}^{2}[m^{2}+{\hat{\beta }}^{2}(-{\hat{\alpha }}^{2}\;total_{2}^{2}+m\;total_{1})\right]}$$ (38)
